# Rotation Matrix Method Based on Ambiguity Function for GNSS Attitude Determination

**DOI:** 10.3390/s16060841

**Published:** 2016-06-08

**Authors:** Yingdong Yang, Xuchu Mao, Weifeng Tian

**Affiliations:** Institute of Navigation and Control, Shanghai Jiao Tong University, Shanghai 201100, China; maoxc@sjtu.edu.cn (X.M.); wftian@sjtu.edu.cn (W.T.)

**Keywords:** GNSS, attitude determination, rotation matrix, AFM method, satellite geometry model, single-frequency

## Abstract

Global navigation satellite systems (GNSS) are well suited for attitude determination. In this study, we use the rotation matrix method to resolve the attitude angle. This method achieves better performance in reducing computational complexity and selecting satellites. The condition of the baseline length is combined with the ambiguity function method (AFM) to search for integer ambiguity, and it is validated in reducing the span of candidates. The noise error is always the key factor to the success rate. It is closely related to the satellite geometry model. In contrast to the AFM, the LAMBDA (Least-squares AMBiguity Decorrelation Adjustment) method gets better results in solving the relationship of the geometric model and the noise error. Although the AFM is more flexible, it is lack of analysis on this aspect. In this study, the influence of the satellite geometry model on the success rate is analyzed in detail. The computation error and the noise error are effectively treated. Not only is the flexibility of the AFM inherited, but the success rate is also increased. An experiment is conducted in a selected campus, and the performance is proved to be effective. Our results are based on simulated and real-time GNSS data and are applied on single-frequency processing, which is known as one of the challenging case of GNSS attitude determination.

## 1. Introduction

Given that global navigation satellite system (GNSS) attitudes are unaffected by drifts and do not require any alignment, GNSS are well suited for attitude determination. The attitude could be noted as yaw (ψ), pitch (θ) and roll (ϕ). One baseline vector composed by two antennas comprises two attitude angles like (ψ，θ). Thus, we could get three attitude angles from two baselines which cannot be arranged in a parallel frame. The basic measurement used for GNSS attitude determination is the phase difference (Δφ) between the signals received by two antennas. However, GNSS attitude determination includes unknown integer ambiguities, which should be solved at first. The baseline length is usually known and it will help in integer ambiguities search. Many approaches have been studied for resolving the GNSS attitude ambiguity resolution problem [1,2,3]. In this study, we use the rotation matrix method (RMM) combined with the AFM method to solve the problem. This method is efficiency used for GNSS ambiguity resolution. When the integer ambiguities are fixed, the attitude angles can be calculated on the basis of the AFM method. Although the AFM is more flexible, it is lack of analysis on the relationship of the geometric model and the noise error. In this study, the influence of the satellite geometry model on the success rate is analyzed. The computation error and the noise error are effectively treated. It is introduced in detail and the performance of this strategy is presented in the sections below. Not only the flexibility of the AFM is inherited, but also the success rate is increased. The results are based on simulated and real-time GNSS data and are applied on single-frequency processing, which is known as one of the challenging case of GNSS attitude determination.

## 2. Model of GNSS Attitude Determination

The carrier-phase equation for GNSS can be written as follows:

(1) $$\varphi =\frac{1}{\lambda }(\rho +\delta \rho +c\delta t_{r}-c\delta t_{s}+\delta \rho_{t}-\delta \rho_{i})-N+\varepsilon$$

where φ is the GNSS receiver carrier-phase observation; λ is the GNSS carrier wavelength; ρ is the range between the receiver antenna and GNSS satellite; δρ is the orbital error along the line of sight from the satellite to station; c is the speed of light; $\delta t_{r}$ is the receiver clock offset from GNSS time; $\delta t_{s}$ is the satellite clock offset from GNSS time; $\delta \rho_{t}$ is the troposphere delay; $\delta \rho_{i}$ is the ionosphere delay; N is the carrier-phase integer ambiguity; and ε is an error term that includes the measurement noise, multi-path errors, others.

An attitude determination system based on GNSS often consists of two receivers to receive the GNSS signals from independent antennae. Given that the common clock is used in the system, the satellite clock error can be removed by a single difference (SD) [4]. For a baseline length of a few meters, the orbital and atmosphere errors in Equation (1) are actually the same, so that these errors can be removed by a single difference. However, the receiver clock error $\delta t_{r}$ still exists. The measurement of the single difference is expressed as:

(2) $$\Delta \varphi =\frac{1}{\lambda }(\Delta \rho +c\Delta \delta t_{r})-\Delta N+\Delta \varepsilon$$

**Figure 1:**
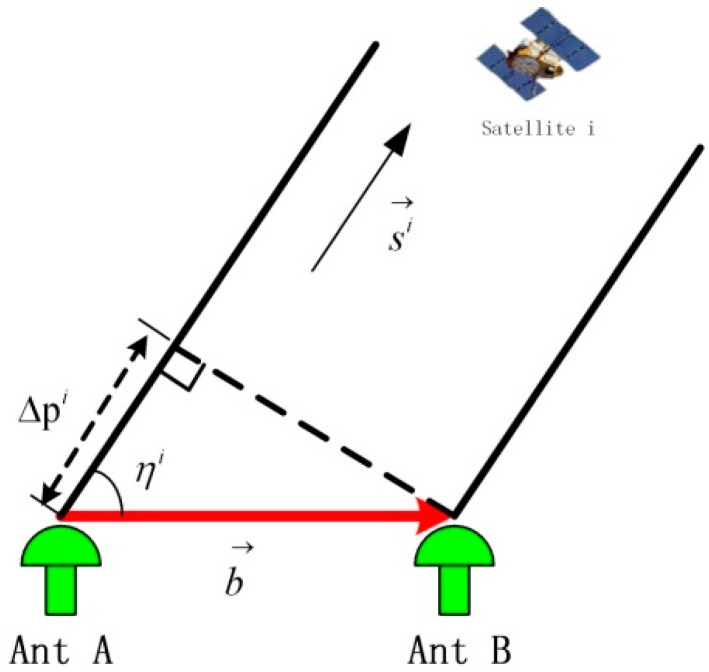
Illustration of the measurement of the SD carrier phase.

As shown in Figure 1, the SD model is built in local level frame (LLF).

**Figure 2:**
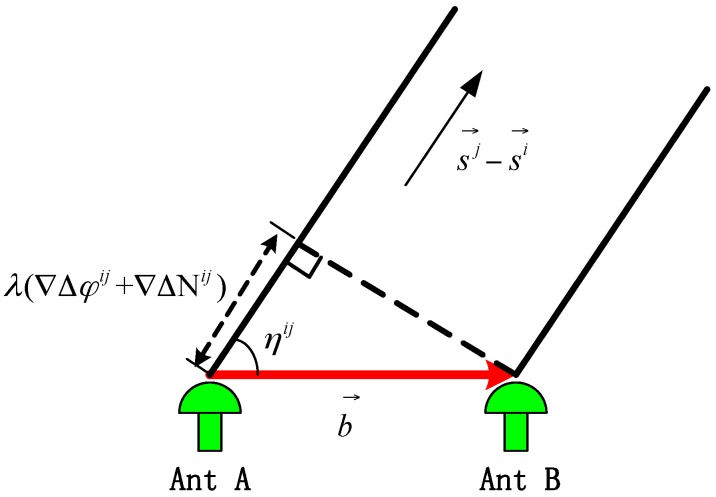
Illustration of the measurement of the DD carrier phase.

In Figure 1, $\vec{b}$ is the baseline vector formed by the antennae A and B, which contains the attitude parameters. The SD carrier phase measurement equation is expressed as:

(3) $$\begin{array}{ll} \lambda (\Delta \varphi^{i}+\Delta N^{i})+c\Delta \delta t_{r} & =\vec{b^{}}\cdot \vec{s^{i}}=\vec{\left|b^{}\right|}\vec{\left|s^{i}\right|}\cos\eta^{i}\\ & =\vec{\left|b^{}\right|}[\sin\beta^{i}\sin\theta +\cos\beta^{i}\cos\theta \cos(\alpha^{i}-\psi )] \end{array}$$

where $\vec{b}=\left|b\right|(\cos\theta \sin\psi ,\cos\theta \cos\psi ,\sin\theta )$ is the baseline vector, ψ, θ are the yaw and pitch, respectively; $\vec{s^{i}}=(\cos\beta^{i}\sin\alpha^{i},\;\cos\beta^{i}\cos\alpha^{i},\sin\beta^{i})$ is the satellite I vector; $\alpha^{i},\;\beta^{i}$ are the yaw and pitch, respectively; $\eta^{i}$ is the included angle between the baseline vector and the satellite I vector; and $\Delta N^{i},\;\Delta \varphi^{i}$ are the integral and decimal part of the SD carrier measurement, respectively. After the measurement of the double difference (DD) between satellites I and J, the receiver clock error can be removed. The DD measurement is expressed as:

(4) $$\begin{array}{l} \lambda (\nabla \Delta \varphi^{ij}+\nabla \Delta N^{ij})=\vec{b^{}}\cdot (\vec{s^{j}}-\vec{s^{i}})=\vec{\;|b^{}|}|\vec{s^{j}}-\vec{s^{i}}|\cos\eta^{ij}\\ \lambda (\nabla \Delta \varphi^{ij}+\nabla \Delta N^{ij})=\vec{\left|b^{}\right|}|\vec{s^{j}}-\vec{s^{i}}|[\sin\beta^{ij}\sin\theta \;+\;\cos\beta^{ij}\cos\theta \cos(\alpha^{ij}-\;\psi )] \end{array}$$

where $\vec{b}=\left|b\right|(\cos\theta \sin\psi ,\;\cos\theta \cos\psi ,\;\sin\theta )$ is the baseline vector, ψ, θ are the yaw and pitch, respectively; $\vec{s^{j}}-\vec{s^{i}}=|\vec{s^{j}}-\vec{s^{i}}|(\cos\beta^{ij}\sin\alpha^{ij},\;\cos\beta^{ij}\cos\alpha^{ij},\;\sin\beta^{ij})$ is the difference between the satellite I and J vectors; $\alpha^{ij},\;\beta^{ij}$ are the yaw and pitch, respectively; $\eta^{ij}$ is the included angle between the baseline vector and the satellite ($\vec{s^{j}}-\vec{s^{i}}$) vector; and $\nabla \Delta N^{ij},\;\nabla \Delta \varphi^{ij}$ are the integral and decimal part of the DD carrier measurement, respectively. As shown in Figure 2, the DD model is built in LLF.

The parameters that are unknown in this function are $\nabla \Delta N^{ij}$, ψ, and θ. Assuming that the value of $\nabla \Delta N^{ij}$ is known in the initial course, we can determine the attitude information using the RMM method. Thus, the first step we should discuss is how to find out the integer ambiguities.

## 3. Rotation Matrix Method in Resolving Equations

The important feature in this method is the use of the RMM to resolve the equations problem. This method is aimed at two equations. Equation (3) is a nonlinear equation, including sine and cosine functions. The solution of the equations is given by the analytical resolution method [5,6], but the solution process is very complex and the error angles of different scales in trigonometric functions have not been analyzed. The rotation matrix method (RMM) is very convenient to obtain. It will be discussed from two aspects.

### 3.1. Basic Model of Space

**Figure 3:**
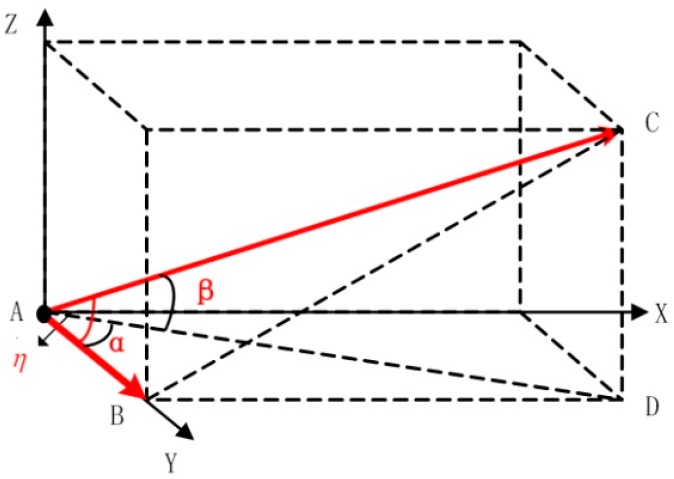
The basic model of spatial geometry.

The basic model of spatial geometry is constructed according to the spatial relationship between the baseline vector and the satellite vector, as shown in Figure 3.

In this figure, $\vec{AB}$ is the baseline vector; $\vec{AC}$ is the satellite vector; β is the pitch of the satellite vector; α is the included angle between the yaw of the baseline vector and the yaw of the satellite vector; and η is the included angle between the baseline vector and the satellite vector. The geometric relations (cosη = cosαcosβ) can be proved in the basic model. This model has a regular structure and a clear geometric relationship. The following operations need to be conducted is to incorporate the actual situation of the baseline vector and the satellite vector into the model.

### 3.2. Generation of Rotation Matrix

Two DD equations are given below:

(5) $$\{\begin{array}{l} \lambda (\nabla \Delta \varphi^{ij}+\nabla \Delta N^{ij})=|\vec{b}||\vec{s^{j}}-\vec{s^{i}}|\cos\eta^{ij}\\ \lambda (\nabla \Delta \varphi^{ik}+\nabla \Delta N^{ik})=|\vec{b}||\vec{s^{k}}-\vec{s^{i}}|\cos\eta^{ik} \end{array}$$

**Figure 4:**
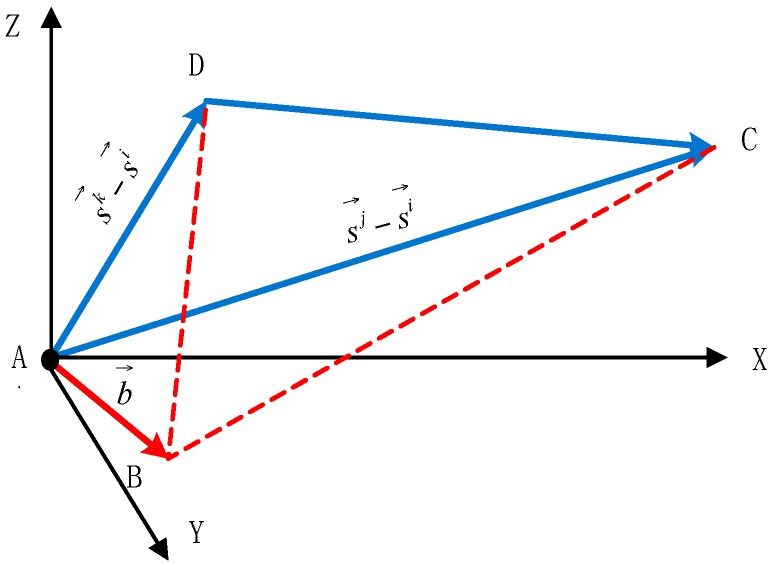
The spatial relation of two DD equations.

The spatial relation of two DD equations is shown in Figure 4.

$$\vec{AC}=\vec{s^{j}}-\vec{s^{i}},\vec{AD}=\vec{s^{k}}-\vec{s^{i}},\vec{AB}=\vec{b},∠BAC=\eta^{ij},∠BAD=\eta^{ik}$$

**Figure 5:**
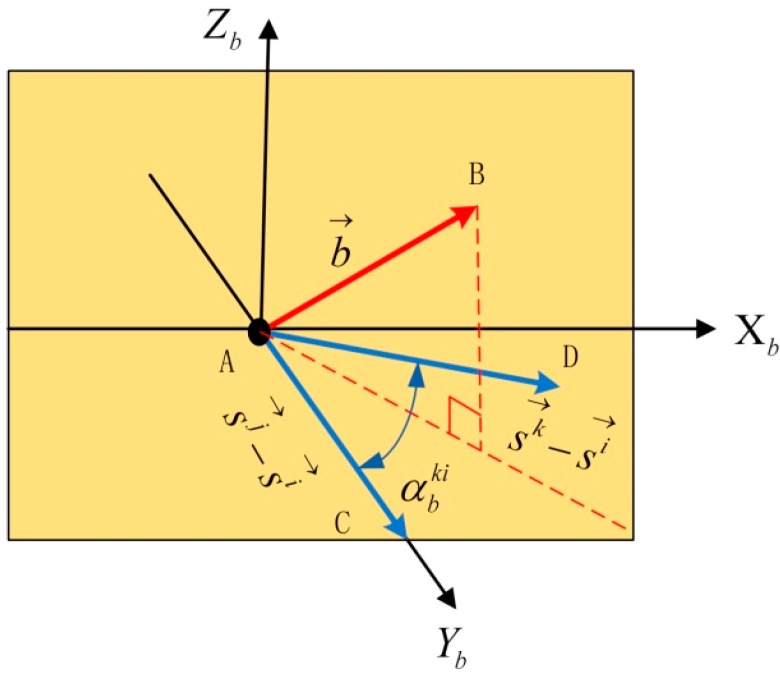
The spatial relation in the new coordinate system.

First, the ACD plane determined by the $\vec{AC}$ vector and the $\vec{AD}$ vector is defined as the level plane of the new coordinate system. Attitude rotation is then conducted. The rotating method is applied from the local level frame (LLF) to the new coordinate system (b):

$$O-X_{n}Y_{n}Z_{n}\frac{Aroud\;Z_{n}\;axis}{rotate\;\alpha }O-X_{1}Y_{1}Z_{1}\frac{Aroud\;X_{1}\;axis}{rotate\;\beta }O-X_{2}Y_{2}Z_{2}\frac{Aroud\;Y_{2}\;axis}{rotate\;\gamma }O-X_{b}Y_{b}Z_{b}$$

where α is the yaw of the $\vec{AC}=\vec{s^{j}}-\vec{s^{i}}$ vector; β is the pitch of the $\vec{AC}=\vec{s^{j}}-\vec{s^{i}}$ vector; γ is the included angle between the ACD plane and the $X_{2}Y_{2}$ plane. In the new coordinate system ($O-X_{b}Y_{b}Z_{b}$), the $\vec{AC}=[0;\;AC_{y};\;0]$ vector and the $\vec{AD}=[AD_{x};\;AD_{y};\;0]$ vector are together in the $X_{b}Y_{b}$ plane. The yaw and pitch of the $\vec{AC}=\vec{s^{j}}-\vec{s^{i}}$ vector are (0, 0), and those of the $\vec{AD}=\vec{s^{k}}-\vec{s^{i}}$ vector are $\left(\alpha_{b}^{ik},\;0\right)$, as shown in Figure 5.

In the new coordinate system ($O-X_{b}Y_{b}Z_{b}$), the baseline vector ($\vec{AB}$) and the satellite vector ($\vec{AC}=\vec{s^{j}}-\vec{s^{i}}$) constitute the basic model of space geometry, the same as the baseline vector ($\vec{AB}$) and the satellite vector ($\vec{AD}=\vec{s^{k}}-\vec{s^{i}}$). At this point, the DD equations are given by:

(6) $$\{\begin{array}{l} \lambda (\nabla \Delta \varphi^{ij}+\nabla \Delta N^{ij})=|\vec{b}||\vec{s^{j}}-\vec{s^{i}}|\cos\theta_{b}\cos\psi_{b}\\ \lambda (\nabla \Delta \varphi^{ik}+\nabla \Delta N^{ik})=|\vec{b}||\vec{s^{k}}-\vec{s^{i}}|\cos\theta_{b}\cos(\psi_{b}-\alpha_{b}^{ik}) \end{array}$$

where $\alpha_{b}^{ik}$ is the yaw of the $\vec{AD}=\vec{s^{k}}-\vec{s^{i}}$ vector in ($O-X_{b}Y_{b}Z_{b}$); $\psi_{b},\theta_{b}$ are the yaw and pitch of the baseline vector in ($O-X_{b}Y_{b}Z_{b}$), respectively. According to Equation (6), the results can be obtained by:

(7) $$\{\begin{array}{l} x_{b}=\frac{A_{2}-A_{1}\cos\alpha_{b}^{ik}}{\sin\alpha_{b}^{ik}}\\ y_{b}=A_{1}\\ z_{b}=\pm \sqrt{|\vec{b}{|}^{2}-x_{b}^{2}-y_{b}^{2}} \end{array},\;\{\begin{array}{l} A_{1}=\frac{\lambda (\nabla \Delta \varphi^{ij}+\nabla \Delta N^{ij})}{\left|\vec{s^{j}}-\vec{s^{i}}\right|}\\ A_{2}=\frac{\lambda (\nabla \Delta \varphi^{ik}+\nabla \Delta N^{ik})}{\left|\vec{s^{k}}-\vec{s^{i}}\right|} \end{array}$$

Finally, the baseline vector $\left(x_{b};\;y_{b};\;z_{b}\right)$ is converted through the rotation matrix from the new coordinate system to the local level frame (LLF). This algorithm reduces the computational complexity compared with the analytical resolution method [5,6]:

(8) $$\left[\begin{array}{c} x\\ y\\ z \end{array}\right]={\left[R_{y}(\gamma )R_{x}(\beta )R_{z}(\alpha )\right]}^{-1}\left[\begin{array}{c} x_{b}\\ y_{b}\\ z_{b} \end{array}\right]$$

### 3.3. Method of Satellite Selection in Resolving Equations

According to Equation (7), the noise effect is larger when the $\left|\vec{s^{x}}-\vec{s^{i}}\right|$ value and the $\alpha_{b}^{ik}$ value are smaller. Thus, the following settings should be applied for satellite selection:

(9) $$\{\begin{array}{c} |\vec{s^{x}}-\vec{s^{i}}|\;>\;0.7\\ |\alpha_{b}^{ik}-90{}^{\circ}|\;\leq \;30{}^{\circ}\;or\;|\alpha_{b}^{ik}-270{}^{\circ}|\;\leq \;30{}^{\circ} \end{array}$$

### 3.4. Integer Ambiguities Determination Method Based on Constraint Conditions

Many solutions have been studied to determine integer ambiguities, and all kinds of constraint conditions exist, such as the fixed baseline or micro-electromechanical system (MEMS) that provides a small-angle search region [7,8]. For the DD equation, an integer ambiguity value that we should first determine, as well as its span, is described as follows:

(10) $$-\frac{\left|\vec{b}||\vec{s^{j}}-\vec{s^{i}}\right|}{\lambda }\leq \nabla \Delta N^{ij}\leq \frac{\left|\vec{b}||\vec{s^{j}}-\vec{s^{i}}\right|}{\lambda }$$

Finally, the integer candidates that pass the constraint conditions are incorporated into the equations, and the candidate solution ($\vec{b}=(x;\;y;\;z)$) can be calculated. These candidate solutions are evaluated and distinguished by the AFM. The AFM was originally proposed by Counselman and Gourevitch and later implemented by Remondi and Mader [9]. The candidate solutions can more easily be determined using RMM directly than by searching for the maximum in the full 2D space. From n pairs of integer ambiguity candidates, m pairs of preliminary solutions exist: $\left(x_{1};\;y_{1};\;z_{1}),(x_{2};\;y_{2};\;z_{2}),\cdots ,(x_{m};\;y_{m};\;z_{m}\right)$. Only one of these solutions is correct, that is, the one that passes via AFM:

(11) $$F(x,\;y,\;z)\;=\;\frac{1}{N-1}\sum_{j=1}^{N-1}\cos2\pi \left\{\nabla \Delta \varphi^{ij}-\frac{\vec{b}\cdot (\vec{s^{j}}-\vec{s^{i})}}{\lambda }\right\}$$

where ($\vec{b}=(x;\;y;\;z)$), N is the number of satellites and (i, j) are the master satellite and the concomitant satellite, respectively. Assuming that m pairs of preliminary solutions $\left(x_{1};y_{1};z_{1}),(x_{2};y_{2};z_{2}),\cdots ,(x_{m};y_{m};z_{m}\right)$ exist in k epoch, the float ambiguity of one group (${\vec{b}}_{p}=(x_{p};\;y_{p};\;z_{p})$) can be described as:

(12) $$\nabla \Delta {\hat{N}}_{p}^{ij}=\nabla \Delta \varphi_{p}^{ij}-\frac{{\vec{b}}_{p}\cdot (\vec{s^{j}}-\vec{s^{i}})}{\lambda }$$

Thus, the ambiguity function is:

(13) $$F_{k}(x_{p},\;y_{p},\;z_{p})\;=\;\frac{1}{N-1}\sum_{j=1}^{N-1}\cos2\pi \nabla \Delta {\hat{N}}_{p}^{ij}$$

**Figure 6:**
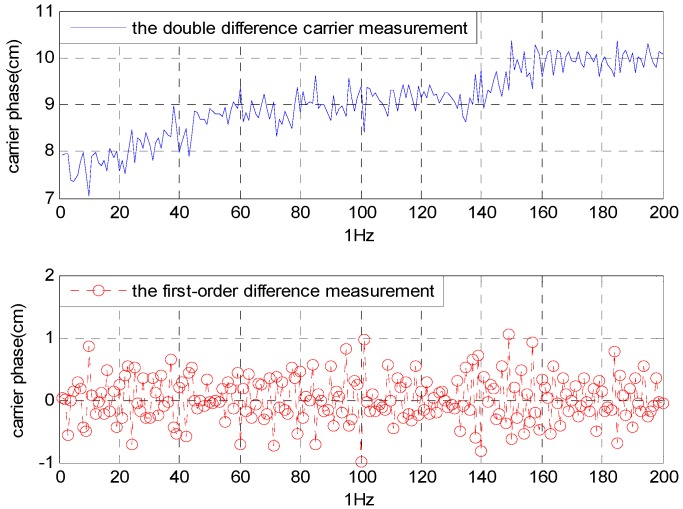
The noise error of the DD carrier measurement.

Considering the measurement noise, a threshold T near 1 is necessary to filter out the incorrect solution [5]. According to the first-order difference of the DD carrier measurement, its fluctuation range is ±0.9 cm (≈0.05 λ) as shown in Figure 6. There are (2*0.05 λ) being introduced in Equation (6). If the noise threshold is set to 0.2 λ, the *T* value is 0.8:

(14) $$F_{k}(x_{p},y_{p},z_{p})\geq T$$

For N satellites tracked, the integer ambiguity vector is described as:

(15) $$\nabla \Delta N_{p}^{ij}\;=\;{\left(\left|\nabla \Delta {\hat{N}}_{p}^{i1}\right|,\;\left|\nabla \Delta {\hat{N}}_{p}^{i2}\right|,\;\ldots ,\;\left|\nabla \Delta {\hat{N}}_{p}^{i(N-1)}\right|\right)}^{T}$$

where |·| denotes a rounding calculation [5].

In the initial course, the influence of the former epochs should be reduced. The solution is given by:

(16) $$\{\begin{array}{l} W_{k}(\nabla \Delta N_{p}^{ij})\;=\;\frac{1}{M}W_{k-1}(\nabla \Delta N_{p}^{ij})\;+\;\frac{M-1}{M}F_{k}(\psi_{p},\;\theta_{p})\\ W_{1}(\nabla \Delta N_{p}^{ij})\;=\;F_{1}(\psi_{p},\;\theta_{p}) \end{array}$$

The M value is the memory decline factor. After a few epochs, two cases always occur: (a) only one candidate satisfies Equation (14) at epoch *k*, or (b) a number of solutions satisfy the threshold. The real value is then selected with the following method. Ideally, for example, the number of satellites is greater than 8, and the geometric relationship is relatively good. The optimal value is obtained when one of the solutions is twice the suboptimal value [6]. The inequality is given by:

(17) $$\frac{W_{k}{\left(\nabla \Delta N_{p}^{ij}\right)}_{optimal}}{W_{k}{\left(\nabla \Delta N_{p}^{ij}\right)}_{suboptimal}}\;>\;2$$

**Figure 7:**
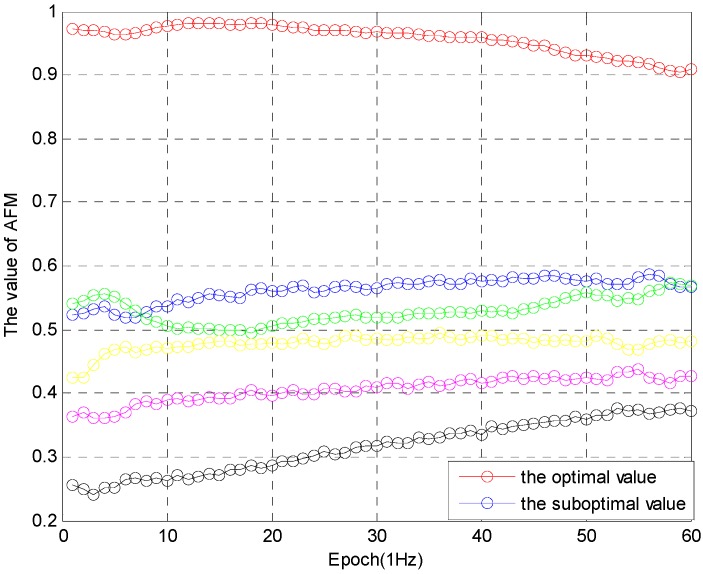
The value ($W_{k}$) of AFM.

In practical application, this ratio is slightly less than 2, while the ratio remains stable. If the optimal value is greater than 0.9, and the difference between the optimal value and the suboptimal value is greater than 0.3, the optimal solution is determined after 50 epochs, as shown in Figure 7:

(18) $$\left\{\begin{array}{c} W_{k}{\left(\nabla \Delta N_{p}^{ij}\right)}_{optimal}>\;0.9\\ W_{k}{\left(\nabla \Delta N_{p}^{ij}\right)}_{optimal}-\;W_{k}{\left(\nabla \Delta N_{p}^{ij}\right)}_{suboptimal}>\;0.3 \end{array},\;k\;>\;50epochs(\mathrm{duration}\right)$$

If the computation error and the noise error are not effectively treated, the success rate of the solution will be decreased. Thus, the influence of the satellite geometry model on the error should be analyzed in detail. In contrast to the AFM, the geometric correlation methods, such as the LAMBDA method [10], the C-LAMBDA method [11,12,13,14] and the M-LAMBDA method [15], get better performance in solving the relationship of the geometric model and the noise error. This relationship about the AFM is analyzed in the sections below. The computation error and the noise error are effectively treated. Not only the flexibility of the AFM is inherited, but also the success rate is increased.

## 4. The Relationship of the Geometric Model and the Noise Error

### 4.1. The Influence of the Noise Error on the DD Equations

For the convenience of analysis, according to Equations (7) and (9), assuming that the satellite parameters of the DD equations are set to $|\vec{s^{j}}-\vec{s^{i}}|\;=\;0.7,\;|\vec{s^{k}}-\vec{s^{i}}|\;=\;0.7,\;\alpha_{b}^{ik}\;=\;70{}^{\circ}$. Now noise is added to Equation (7) and the parameters are substituted into Equation (7):

(19) $$\{\begin{array}{l} x_{b}=\;1.519\;*\;\lambda [(\nabla \Delta \varphi^{ik}+\nabla \Delta N^{ik}+\varepsilon^{ik})-0.342*(\nabla \Delta \varphi^{ij}+\nabla \Delta N^{ij}+\varepsilon^{ij})]\\ y_{b}=\;1.428\;*\;\lambda (\nabla \Delta \varphi^{ij}+\nabla \Delta N^{ij}+\varepsilon^{ij})\\ z_{b}=\;\pm \sqrt{|\vec{b}{|}^{2}-\;x_{b}^{2}-\;y_{b}^{2}} \end{array}$$

Assuming that the vector ($X_{b},\;Y_{b},\;Z_{b}$) is the real value of the vector ($\vec{b}\;=\;(x_{b};\;y_{b};\;z_{b})$), Equation (19) can be expressed as:

(20) $$\{\begin{array}{l} x_{b}=\;X_{b}+\;1.519\lambda {\left(\varepsilon^{ik}-0.342\varepsilon^{ij}\right)}_{}\\ y_{b}=\;Y_{b}+\;1.428\lambda \varepsilon^{ij}\\ z_{b}=\;\pm \sqrt{|\vec{b}{|}^{2}-\;x_{b}^{2}-\;y_{b}^{2}} \end{array}$$

According to Equation (4), although the DD model eliminates the receiver clock error and the satellite clock error, the cost of the DD measurement noise root mean square error is $\sqrt{2}$ times than the SD measurement, which is generally about 1 cm (*i.e.*, roughly 0.05 GPS L1wavelength). Thus, according to Equation (20), the noise root mean square error ($rmse(\vec{b})$) of the baseline vector ($\vec{b}\;=\;(x_{b};\;y_{b};\;z_{b})$) is expressed as:

(21) $$\{\begin{array}{l} rmse(x_{b})\;=\;0.102\;\lambda \\ rmse(y_{b})\;=\;0.071\;\lambda \\ rmse(z_{b})\;\approx \;0.173\;\lambda \end{array}$$

Now, according to Equation (21), the noise error of the ambiguity function (F(x, y, z)) is analyzed:

(22) $$F(x,\;y,\;z)\;=\;\frac{1}{N-1}\sum_{j=1}^{N-1}\cos2\pi \left\{\nabla \Delta \varphi^{ij}-\;\frac{\vec{b}\cdot (\vec{s^{m}}-\vec{s^{i})}}{\lambda }\right\}$$

For the convenience of analysis, the satellite vector ($\vec{s^{m}}-\vec{s^{i}}$) of the other DD equation is converted through the rotation matrix from the local level frame (LLF) to the new coordinate system ($O-X_{b}Y_{b}Z_{b}$):

(23) $$[\begin{array}{l} \vec{(s^{m}}-\vec{s^{i}{)}_{b(x)}}\\ \vec{(s^{m}}-\vec{s^{i}{)}_{b(y)}}\\ \vec{(s^{m}}-\vec{s^{i}{)}_{b(z)}} \end{array}]=[R_{y}(\gamma )R_{x}(\beta )R_{z}(\alpha )]\left[\begin{array}{l} \vec{(s^{m}}-\vec{s^{i}{)}_{LLF(x)}}\\ \vec{(s^{m}}-\vec{s^{i}{)}_{LLF(y)}}\\ \vec{(s^{m}}-\vec{s^{i}{)}_{LLF(z)}} \end{array}\right]$$

Then, Equation (23) is substituted into Equation (12) and the result is expressed as:

(24) $$\begin{array}{l} \nabla \Delta {\hat{N}}^{im}=\;\nabla \Delta \varphi^{im}+\;\varepsilon^{im}-\;\frac{{\left(\vec{s^{m}}-\vec{s^{i}}\right)}_{b}\cdot {{(x}_{b}{,y}_{b}{,z}_{b})}^{T}}{\lambda }\\ \nabla \Delta {\hat{N}}^{im}=\;\nabla \Delta \varphi^{im}+\;\varepsilon^{im}-\;\frac{{\left(\vec{s^{m}}-\vec{s^{i}}\right)}_{b(x)}\cdot x_{b}+\;{\left(\vec{s^{m}}-\vec{s^{i}}\right)}_{b(y)}\cdot y_{b}+\;{\left(\vec{s^{m}}-\vec{s^{i}}\right)}_{b(z)}\cdot z_{b}}{\lambda } \end{array}$$

where $\varepsilon^{im}$ is the noise error of the DD equation (the satellite vector: $\vec{s^{m}}-\vec{s^{i}}$); $\nabla \Delta {\hat{N}}^{im}$ is the float ambiguity. The Equation (21) is substituted into Equation (24), the noise root mean square error ($rmse(\nabla \Delta {\hat{N}}^{im})$) of the float ambiguity is expressed as:

(25) $$\begin{array}{l} rmse(\nabla \Delta {\hat{N}}^{im})\;=\mathrm{rmse}(\varepsilon^{im})+\;\frac{{\left(\vec{s^{m}}-\vec{s^{i}}\right)}_{b}\cdot rmse{\left(x_{b},y_{b},z_{b}\right)}^{T}}{\lambda }\\ rmse(\nabla \Delta {\hat{N}}^{im})\;\approx \;0.05\;+\;{\left(\vec{s^{m}}-\vec{s^{i}}\right)}_{b(x)}\cdot 0.102\;+\;{\left(\vec{s^{m}}-\vec{s^{i}}\right)}_{b(y)}\cdot 0.071\;+\;{\left(\vec{s^{m}}-\vec{s^{i}}\right)}_{b(z)}\cdot 0.173 \end{array}$$

### 4.2. Ambiguity Decorrelation Adjustment of the Geometric Relationship

According to the previous analysis, if the basic equations (Equation (6)) of the DD model are determined, the satellite parameters ($\vec{s^{j}}-\vec{s^{i}},\vec{s^{k}}-\vec{s^{i}},\;\alpha_{b}^{ik}$) are also determined. The method for satellite selection is based on Equation (9). According to Equation (25), the value ($rmse(\nabla \Delta {\hat{N}}^{im})$) of the float ambiguity is only related to the other satellite vector ${\left(\vec{s^{m}}-\vec{s^{i}}\right)}_{b}$ and the candidate vector ($\vec{b}\;=\;(x_{b};\;y_{b};\;z_{b})$). It represents the geometric relationship between the candidate vector and the satellite vector. If the correlation of geometric relationship is smaller, the value ($rmse(\nabla \Delta {\hat{N}}^{im})$) of the float ambiguity is smaller. Thus, we need to find the suitable satellite vector ($\vec{s^{m}}-\vec{s^{x}}$), so that the value ($rmse(\nabla \Delta {\hat{N}}^{im})$) of the float ambiguity is the smallest. This process is equivalent to ambiguity decorrelation adjustment of the LAMBDA method [10,11,12]. If this value ($rmse(\nabla \Delta {\hat{N}}^{im})$) is smaller, the correlation interference of the noise error is smaller and the robustness of the ambiguity function (F(x, y, z)) is better. In contrast to the LAMBDA method, this method gets better performance in reducing computational complexity.

### 4.3. Comparison of the Simulation Results

**Figure 8:**
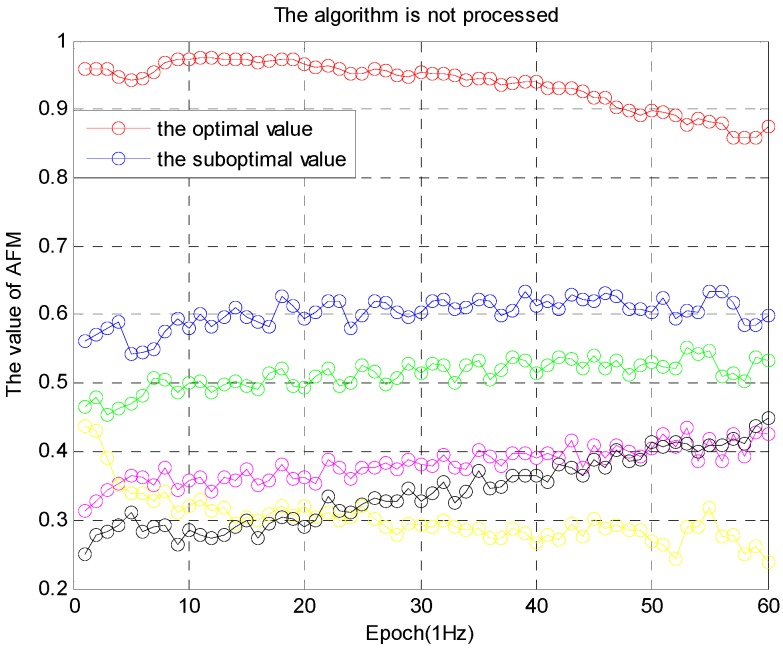
Algorithm after not-processing.

**Figure 9:**
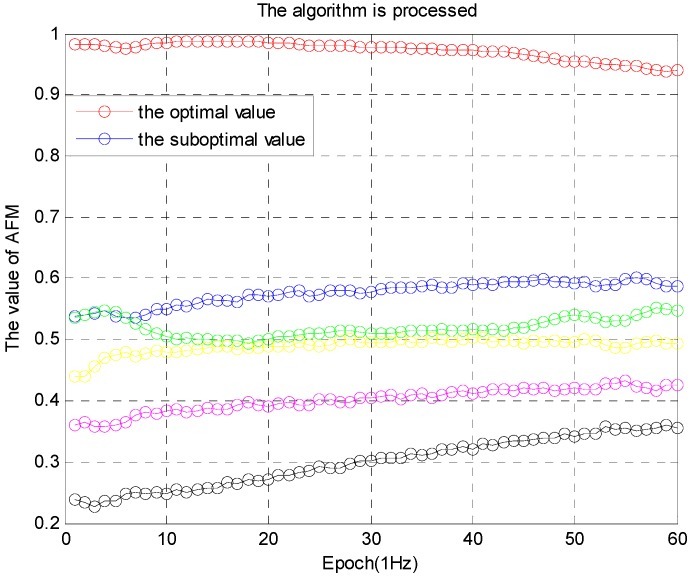
Algorithm after processing.

The simulation is performed from two aspects. In the first aspect, the algorithm is processed with ambiguity decorrelation adjustment of the noise error, and the other one is not do it. The simulation results are shown in Figure 8 and Figure 9.

The performance of the algorithm in Figure 9 is better than the algorithm in Figure 8. The algorithm is effective, and the robustness of the ambiguity function (F(x,y,z)) is improved. In the second aspect, the success rate of processing and not-processing is compared, as shown in Table 1.

## 5. Experimental Attitude Determination Results

**Figure 10:**
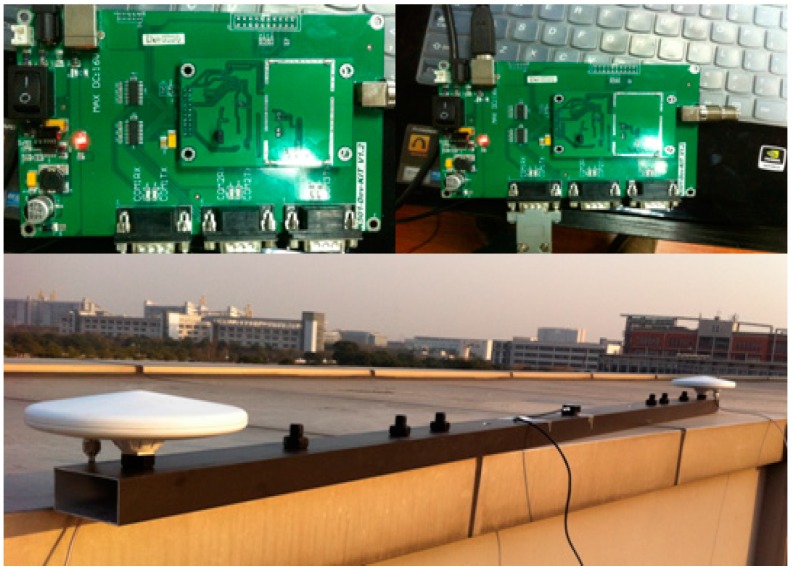
Receiver electric circuit and antennae configuration.

In this section the RMM method is demonstrated using data collected in an experiment. The experiment is conducted on the top of a building. In this experiment, two receivers (COMNAV K500, Shanghai Siyue Technology Co. Ltd, Shanghai, China) are used, and both are connected to the GNSS antennae. The receiver electro-circuit is shown in Figure 10.

**Figure 11:**
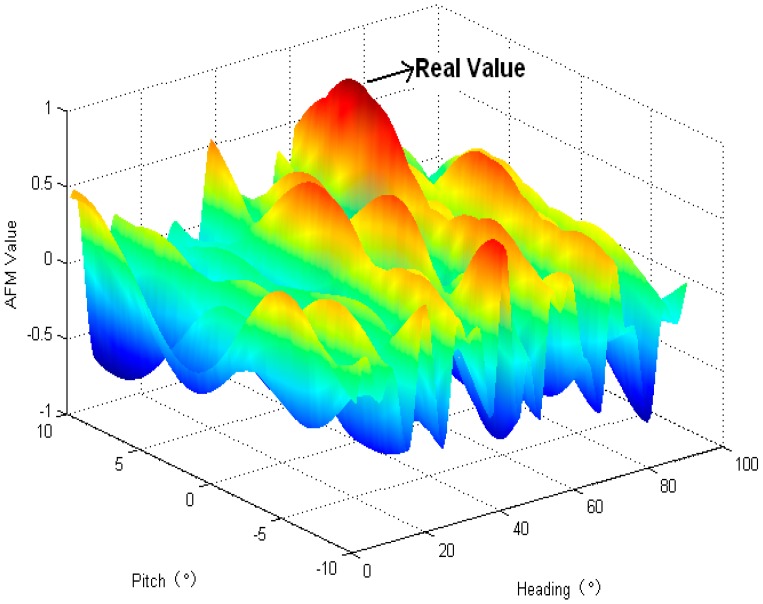
Adaptive function value demonstration.

The baseline is constrained with 2.0 m and 0.5 m and the pitch angle search region of 20 degrees is provided by MEMS. During the initial step, the integer ambiguity combination is resolved by RMM method and the real value is work out after a few epochs, as is shown in Figure 11, and the real value is a point on the peak obviously.

**Figure 12:**
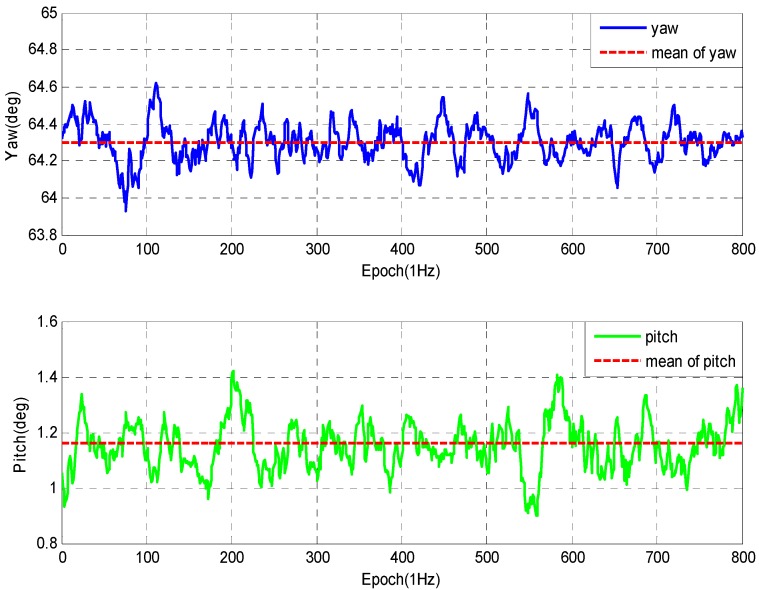
Experimental data of 1st group.

**Figure 13:**
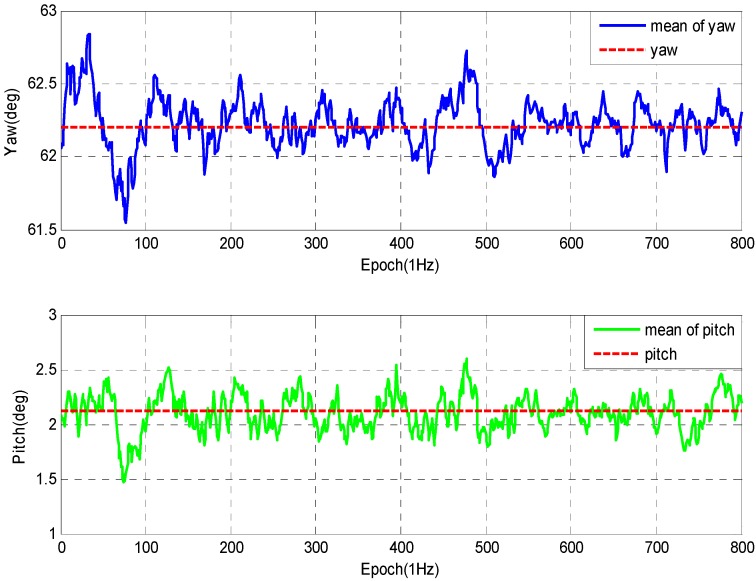
Experimental data of 2nd group.

In this test, the number of locked GPS satellites is about eight, and the geometry of observations for this test is reasonably good. The experiment is performed from two groups in different arrangement. In Figure 12, with 2.0 m baseline, the standard deviation of the yaw and pitch angles are about 0.14° (1σ) and 0.18° (1σ), while with 0.5 m baseline the standard deviation reaches about 0.2° (1σ) and 0.25° (1σ) as shown in Figure 13. However, the calculation is reduced greatly.

The performance is good with high accuracy shown in Table 2.

## 6. Conclusions

This study describes the rotation matrix method and the relationship of the geometric model and the noise error for single-frequency and single-epoch GNSS attitude determination. The rotation matrix method reduces the computational complexity compared with the analytical resolution method [5,6]. In RMM, the calculation is reduced greatly and the error angles of different scales in trigonometric functions are effectively avoided. In contrast to the AFM, the geometric correlation methods [10,11,12,13,14,15] get better performance in solving the relationship of the geometric model and the noise error. Although the AFM is more flexible, there is a lack of analysis on this aspect. In the study, this relationship about the AFM is analyzed in detail. The computation error and the noise error are effectively treated. Not only is the flexibility of the AFM inherited, but the success rate is also increased. According to our simulations and real-time experiments, this method is verified as very reliable and effective. The computational complexity is greatly reduced and the success rate is effectively increased. In future studies, we plan to combine the method with an inertial navigation system (INS) for tight combination.

## Figures and Tables

**Table 1:** Single-frequency, single-epoch, GNSS ambiguity success rates based on Equation (17) and Equation (18) for the processing (Y) and not-processing (N) RMM-AFM methods.

|b| = 200 cm	Carrier Phase Precision: 3.0 mm		Pseudorange Precision: 15 cm	
#Sats	Method	Success Rate (%)	Method	Success Rate (%)
5	Y	72.1	N	43.1
6	Y	92.4	N	67.3
7	Y	95.1	N	77.4
8	Y	97.3	N	82.1

**Table 2:** Standard errors of attitude determination experiment.

Test	Epochs	Baseline (m)	Attitude (°)	Std. Errors
1	800	2.00	Yaw	0.1483
			Pitch	0.1852
2	500	2.00	Yaw	0.1503
			Pitch	0.1801
3	800	0.5	Yaw	0.2121
			Pitch	0.2509
4	500	0.5	Yaw	0.2221
			Pitch	0.2615
